# Prognostic factors for chronic arthritis in children with acute joint swelling

**DOI:** 10.1186/1546-0096-12-S1-P51

**Published:** 2014-09-17

**Authors:** Skirmante Rusoniene, Violeta Panaviene, Audrone Eidukaite, Marija Jakutovic

**Affiliations:** 1 Children Hospital, Affiliate of Vilnius University Hospital Santariskiu Klinikos, Vilnius, Lithuania

## Introduction

Acute inflammatory arthritis is frequent clinical sign in children with variable outcomes. Often post infectious or viral arthritis progress to chronic joint disease

## Objectives

Our aim is to determine prognostic factors that predict the course of chronic arthritis in children.

## Methods

116 consecutive children with acute arthritis with symptoms duration < 6 weeks included in prospective study. A standardize rheumatologic evaluation was performed on newly referred patients. Possible diagnostic variables collected at the first visit: active joints count, symptoms duration, time of morning stiffness, erythrocyte sedimentation rate (ESR), C reactive protein (SRP), antinuclear antibodies (ANA), HLA B27, myeloid-related protein 8/14 (MRP8/14) and IL-6 . Arthritis outcome was defined at 1 and 2 years follow-up. We considered the clinical outcomes - chronic arthritis and inactive disease. Multiple linear regression with forward stepwise was used to determine the prognostic variables.

## Results

109/116 patients completed follow –up period. Of all patients 36,7 % had clinically active (or chronic) arthritis after 1 year, and 30 % - after 2 year follow-up periods, and had been treated with appropriate therapy after establishing initial diagnose (JIA, arthritis related to infections and self-limited undifferentiated arthritis). The mean serum level of MRP8/14 at baseline measured in patients with arthritis related to infection was 11233,74 ng/ml, and 7836,05 ng/ml in JIA patients, compared with self-limiting arthritis – 4832,19 ng/ml (p<0,001). Predominantly very high MRP8/14 concentrations were measured in patients who developed chronic disease.

Logistic regression analysis showed that significant predictors of chronic disease were presence of morning stiffness (OR 8,7 [2,15-35,04]), arthritis in ≥ 5 joints(OR 6,3 [2-19,9]) and MRP8/14 concentration >5785 ng/ml (OR 4,4 [1,59-12,01]) at baseline.

## Conclusion

The early presence of morning stiffness, polyarthritic joint involvement and high MRP8/14 concentration in a child with acute arthritis indicates the likelihood of chronic disease after 1 and 2 years follow-up.

## Disclosure of interest

None declared.

